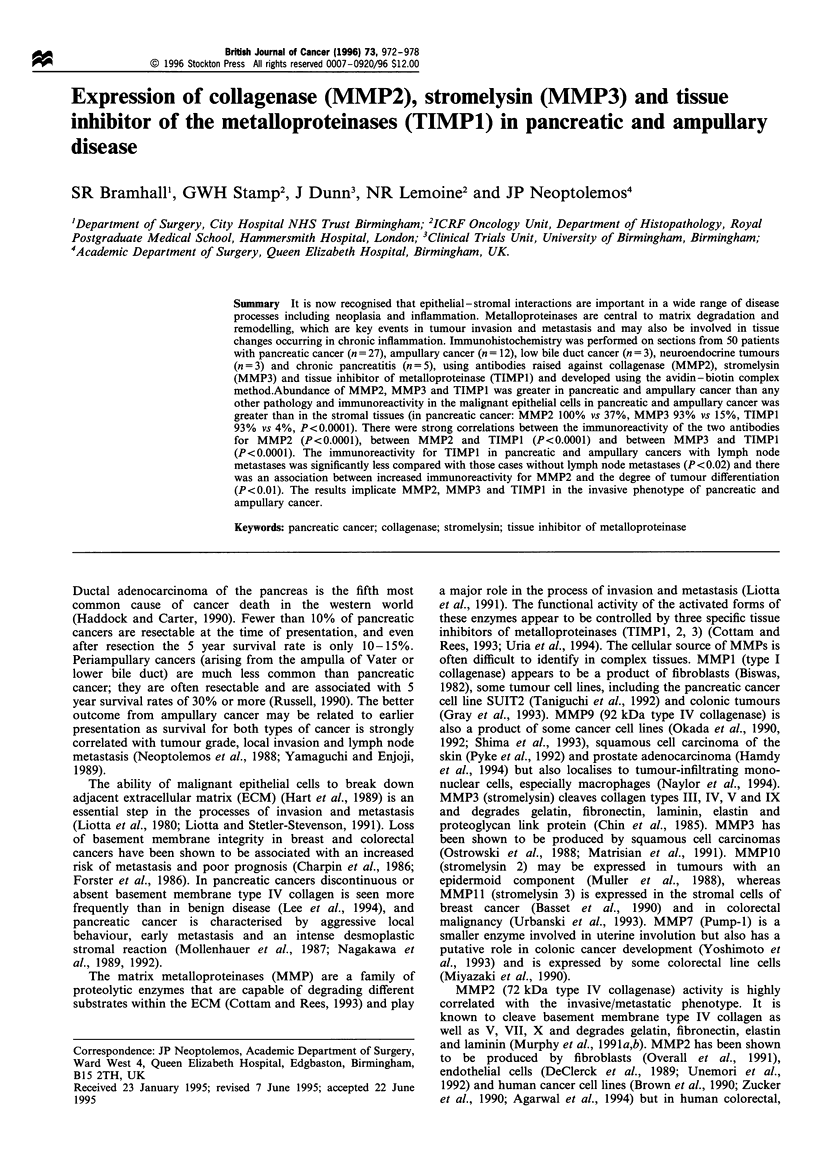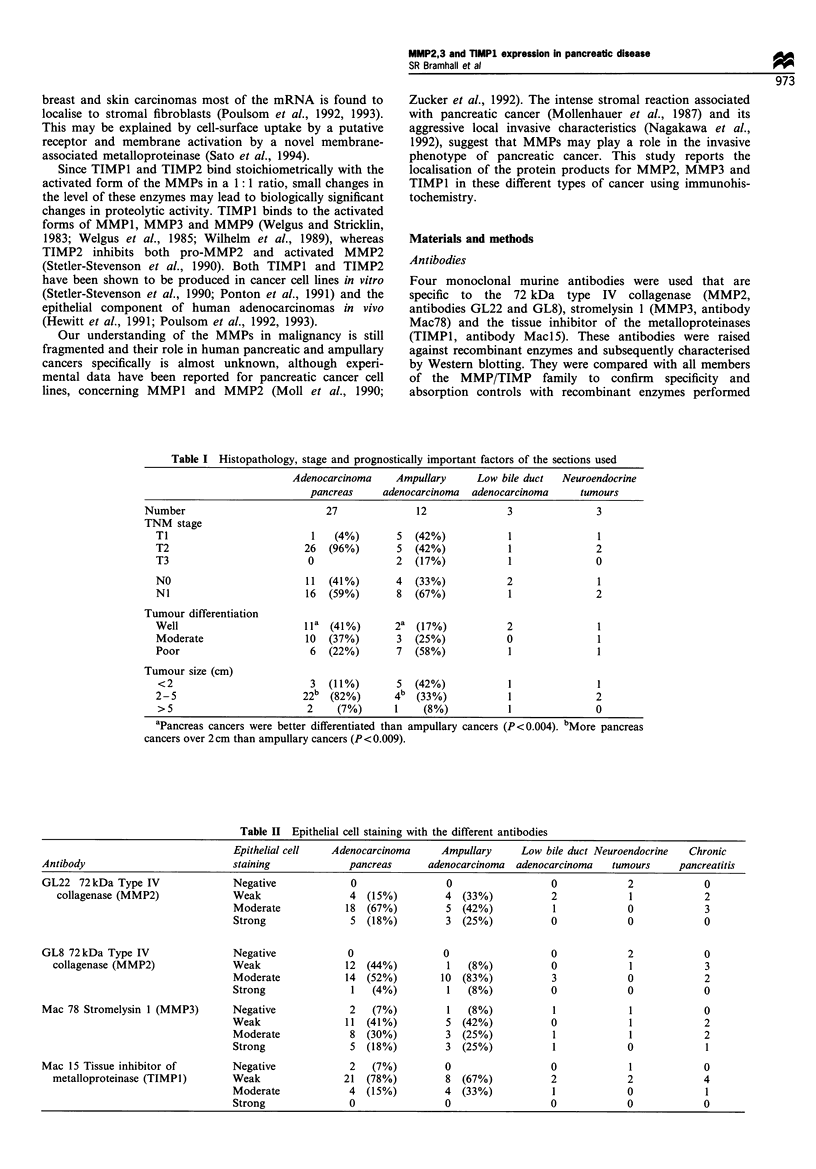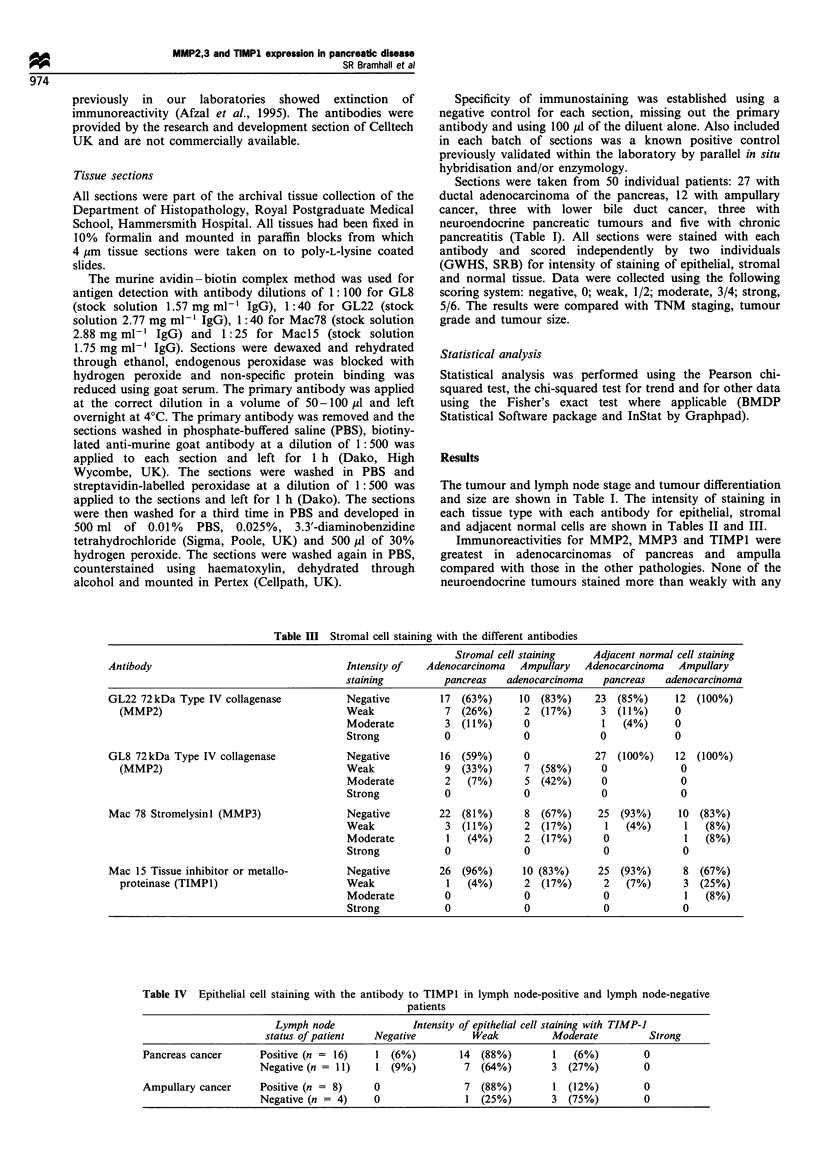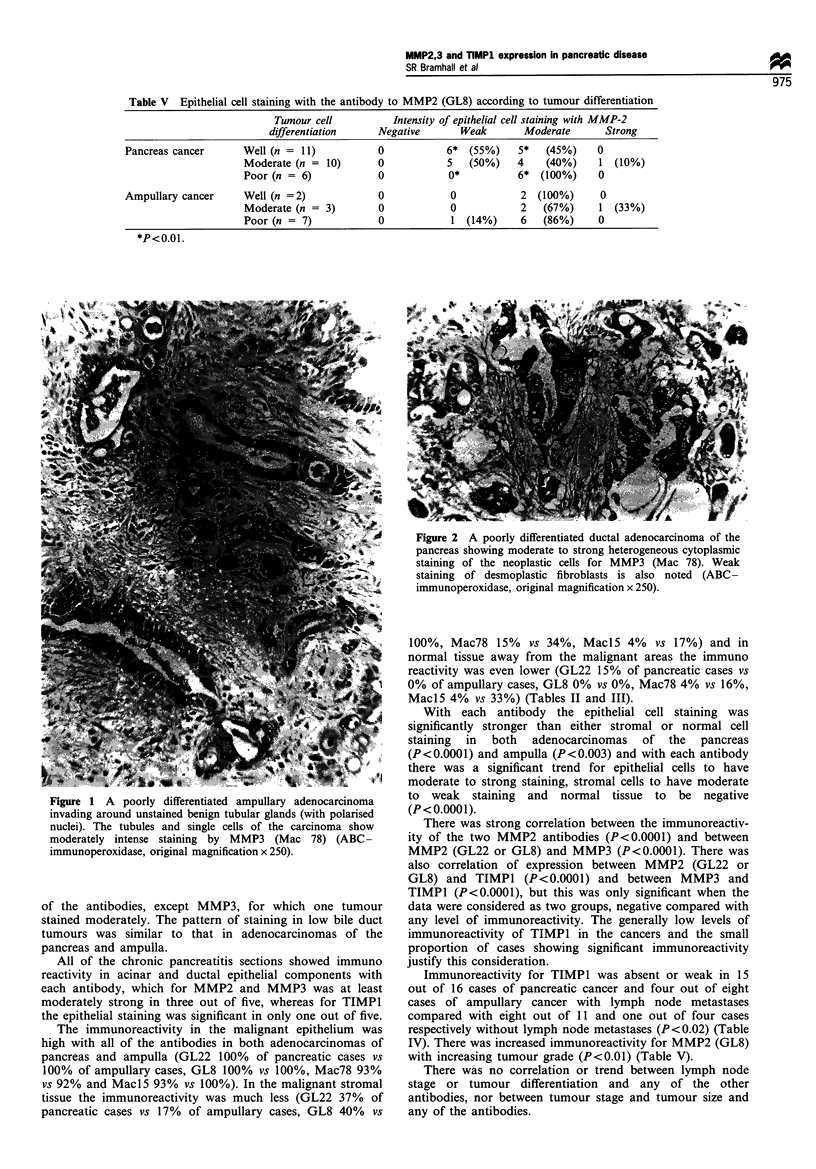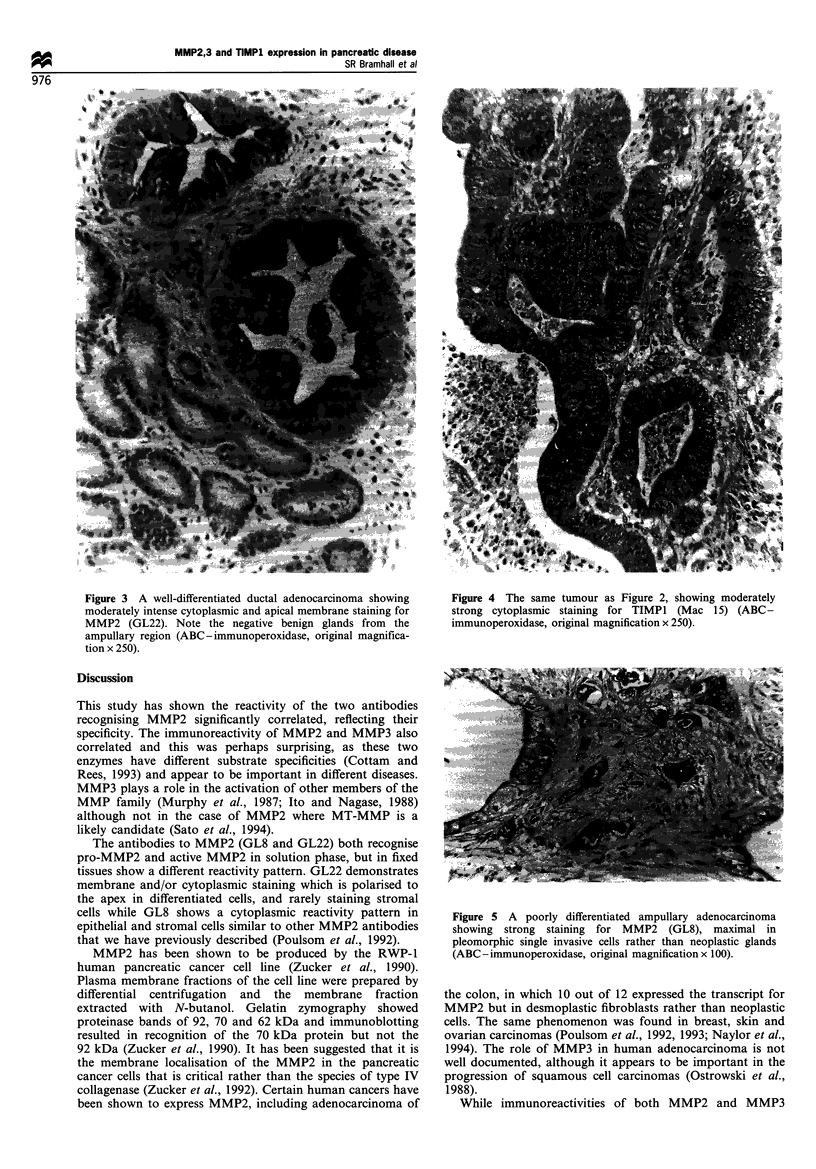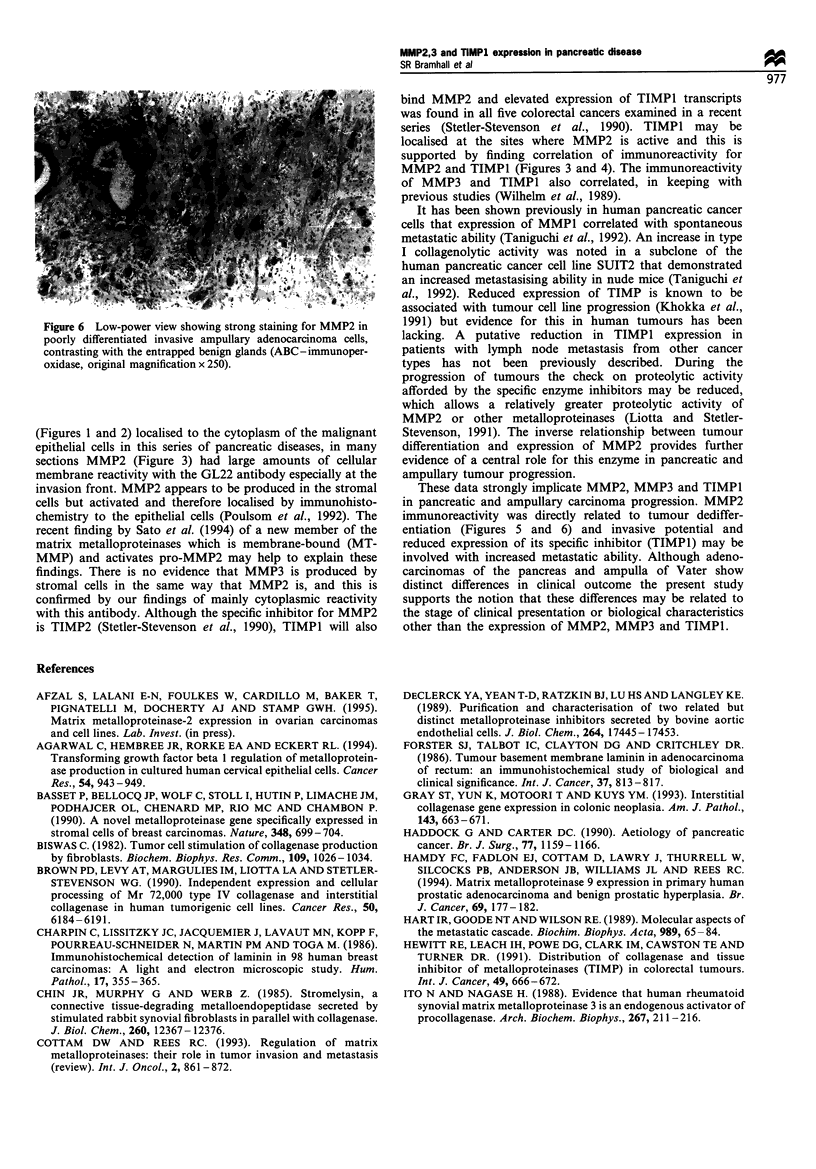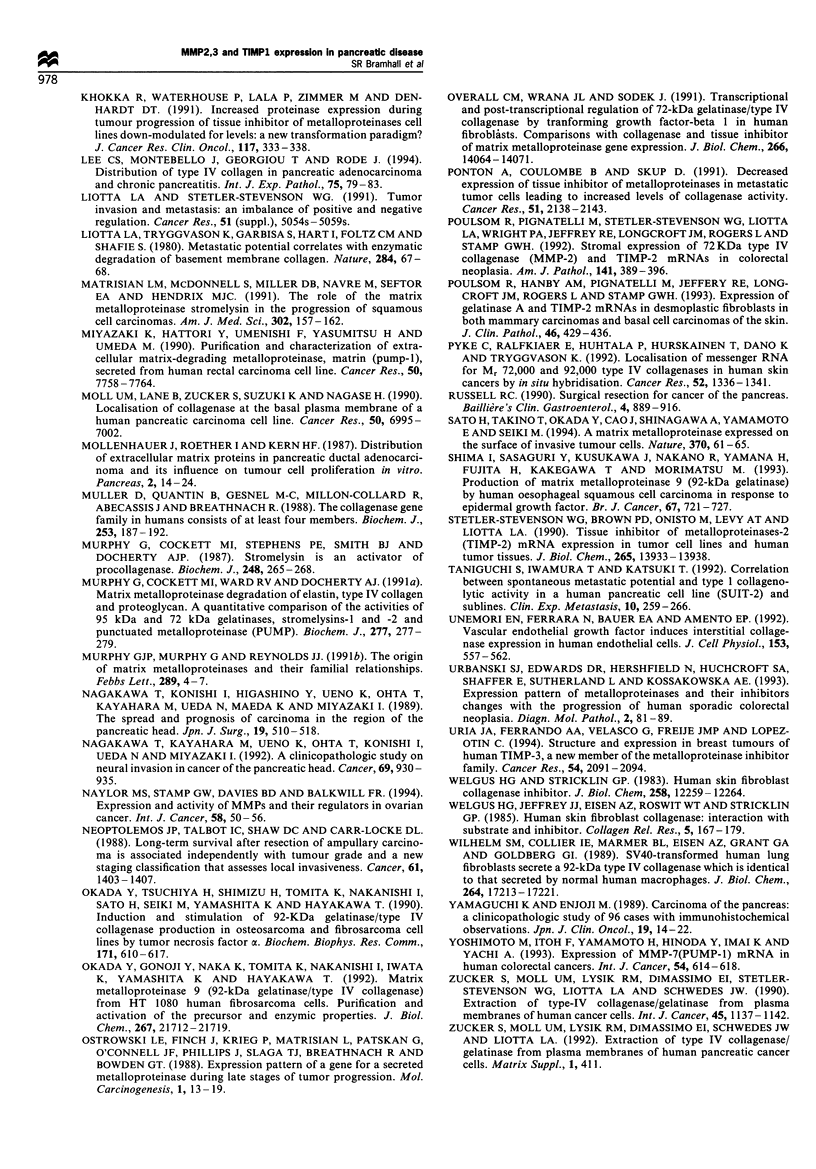# Expression of collagenase (MMP2), stromelysin (MMP3) and tissue inhibitor of the metalloproteinases (TIMP1) in pancreatic and ampullary disease.

**DOI:** 10.1038/bjc.1996.190

**Published:** 1996-04

**Authors:** S. R. Bramhall, G. W. Stamp, J. Dunn, N. R. Lemoine, J. P. Neoptolemos

**Affiliations:** Department of Surgery, City Hospital NHS Trust Birmingham, UK.

## Abstract

**Images:**


British Journal of Cancer (1996) 73, 972-978
© 1996 Stockton Press All rights reserved 0007-0920/96 $12.00

### Expression of collagenase (MMP2), stromelysin (MMP3) and tissue inhibitor of the metalloproteinases (TIMP1) in pancreatic and ampullary disease

SR Bramhall[1], GWH Stamp[2], J Dunn[3], NR Lemoine[2] and JP Neoptolemos[4]

[1]*Department of Surgery, City Hospital NHS Trust Birmingham;* [2]*ICRF Oncology Unit, Department of Histopathology, Royal Postgraduate Medical School, Hammersmith Hospital, London;* [3]*Clinical Trials Unit, University of Birmingham, Birmingham;* [4]*Academic Department of Surgery, Queen Elizabeth Hospital, Birmingham, UK.*

**Summary** It is now recognised that epithelial–stromal interactions are important in a wide range of disease processes including neoplasia and inflammation. Metalloproteinases are central to matrix degradation and remodelling, which are key events in tumour invasion and metastasis and may also be involved in tissue changes occurring in chronic inflammation. Immunohistochemistry was performed on sections from 50 patients with pancreatic cancer ($n=27$), ampullary cancer ($n=12$), low bile duct cancer ($n=3$), neuroendocrine tumours ($n=3$) and chronic pancreatitis ($n=5$), using antibodies raised against collagenase (MMP2), stromelysin (MMP3) and tissue inhibitor of metalloproteinase (TIMP1) and developed using the avidin–biotin complex method.Abundance of MMP2, MMP3 and TIMP1 was greater in pancreatic and ampullary cancer than any other pathology and immunoreactivity in the malignant epithelial cells in pancreatic and ampullary cancer was greater than in the stromal tissues (in pancreatic cancer: MMP2 100% *vs* 37%, MMP3 93% *vs* 15%, TIMP1 93% *vs* 4%, $P<0.0001$). There were strong correlations between the immunoreactivity of the two antibodies for MMP2 ($P<0.0001$), between MMP2 and TIMP1 ($P<0.0001$) and between MMP3 and TIMP1 ($P<0.0001$). The immunoreactivity for TIMP1 in pancreatic and ampullary cancers with lymph node metastases was significantly less compared with those cases without lymph node metastases ($P<0.02$) and there was an association between increased immunoreactivity for MMP2 and the degree of tumour differentiation ($P<0.01$). The results implicate MMP2, MMP3 and TIMP1 in the invasive phenotype of pancreatic and ampullary cancer.

**Keywords:** pancreatic cancer; collagenase; stromelysin; tissue inhibitor of metalloproteinase

Ductal adenocarcinoma of the pancreas is the fifth most common cause of cancer death in the western world (Haddock and Carter, 1990). Fewer than 10% of pancreatic cancers are resectable at the time of presentation, and even after resection the 5 year survival rate is only 10–15%. Periampullary cancers (arising from the ampulla of Vater or lower bile duct) are much less common than pancreatic cancer; they are often resectable and are associated with 5 year survival rates of 30% or more (Russell, 1990). The better outcome from ampullary cancer may be related to earlier presentation as survival for both types of cancer is strongly correlated with tumour grade, local invasion and lymph node metastasis (Neoptolemos *et al.*, 1988; Yamaguchi and Enjoji, 1989).

The ability of malignant epithelial cells to break down adjacent extracellular matrix (ECM) (Hart *et al.*, 1989) is an essential step in the processes of invasion and metastasis (Liotta *et al.*, 1980; Liotta and Stetler-Stevenson, 1991). Loss of basement membrane integrity in breast and colorectal cancers have been shown to be associated with an increased risk of metastasis and poor prognosis (Charpin *et al.*, 1986; Forster *et al.*, 1986). In pancreatic cancers discontinuous or absent basement membrane type IV collagen is seen more frequently than in benign disease (Lee *et al.*, 1994), and pancreatic cancer is characterised by aggressive local behaviour, early metastasis and an intense desmoplastic stromal reaction (Mollenhauer *et al.*, 1987; Nagakawa *et al.*, 1989, 1992).

The matrix metalloproteinases (MMP) are a family of proteolytic enzymes that are capable of degrading different substrates within the ECM (Cottam and Rees, 1993) and play a major role in the process of invasion and metastasis (Liotta *et al.*, 1991). The functional activity of the activated forms of these enzymes appear to be controlled by three specific tissue inhibitors of metalloproteinases (TIMP1, 2, 3) (Cottam and Rees, 1993; Uria *et al.*, 1994). The cellular source of MMPs is often difficult to identify in complex tissues. MMP1 (type I collagenase) appears to be a product of fibroblasts (Biswas, 1982), some tumour cell lines, including the pancreatic cancer cell line SUIT2 (Taniguchi *et al.*, 1992) and colonic tumours (Gray *et al.*, 1993). MMP9 (92 kDa type IV collagenase) is also a product of some cancer cell lines (Okada *et al.*, 1990, 1992; Shima *et al.*, 1993), squamous cell carcinoma of the skin (Pyke *et al.*, 1992) and prostate adenocarcinoma (Hamdy *et al.*, 1994) but also localises to tumour-infiltrating mononuclear cells, especially macrophages (Naylor *et al.*, 1994). MMP3 (stromelysin) cleaves collagen types III, IV, V and IX and degrades gelatin, fibronectin, laminin, elastin and proteoglycan link protein (Chin *et al.*, 1985). MMP3 has been shown to be produced by squamous cell carcinomas (Ostrowski *et al.*, 1988; Matrisian *et al.*, 1991). MMP10 (stromelysin 2) may be expressed in tumours with an epidermoid component (Muller *et al.*, 1988), whereas MMP11 (stromelysin 3) is expressed in the stromal cells of breast cancer (Basset *et al.*, 1990) and in colorectal malignancy (Urbanski *et al.*, 1993). MMP7 (Pump-1) is a smaller enzyme involved in uterine involution but also has a putative role in colonic cancer development (Yoshimoto *et al.*, 1993) and is expressed by some colorectal line cells (Miyazaki *et al.*, 1990).

MMP2 (72 kDa type IV collagenase) activity is highly correlated with the invasive/metastatic phenotype. It is known to cleave basement membrane type IV collagen as well as V, VII, X and degrades gelatin, fibronectin, elastin and laminin (Murphy *et al.*, 1991*a*,*b*). MMP2 has been shown to be produced by fibroblasts (Overall *et al.*, 1991), endothelial cells (DeClerck *et al.*, 1989; Unemori *et al.*, 1992) and human cancer cell lines (Brown *et al.*, 1990; Zucker *et al.*, 1990; Agarwal *et al.*, 1994) but in human colorectal,

Correspondence: JP Neoptolemos, Academic Department of Surgery, Ward West 4, Queen Elizabeth Hospital, Edgbaston, Birmingham, B15 2TH, UK
Received 23 January 1995; revised 7 June 1995; accepted 22 June 1995

breast and skin carcinomas most of the mRNA is found to localise to stromal fibroblasts (Poulsom et al., 1992, 1993). This may be explained by cell-surface uptake by a putative receptor and membrane activation by a novel membrane-associated metalloproteinase (Sato et al., 1994).

Since TIMP1 and TIMP2 bind stoichiometrically with the activated form of the MMPs in a 1 : 1 ratio, small changes in the level of these enzymes may lead to biologically significant changes in proteolytic activity. TIMP1 binds to the activated forms of MMP1, MMP3 and MMP9 (Welgus and Stricklin, 1983; Welgus et al., 1985; Wilhelm et al., 1989), whereas TIMP2 inhibits both pro-MMP2 and activated MMP2 (Stetler-Stevenson et al., 1990). Both TIMP1 and TIMP2 have been shown to be produced in cancer cell lines *in vitro* (Stetler-Stevenson et al., 1990; Ponton et al., 1991) and the epithelial component of human adenocarcinomas *in vivo* (Hewitt et al., 1991; Poulsom et al., 1992, 1993).

Our understanding of the MMPs in malignancy is still fragmented and their role in human pancreatic and ampullary cancers specifically is almost unknown, although experimental data have been reported for pancreatic cancer cell lines, concerning MMP1 and MMP2 (Moll et al., 1990; Zucker et al., 1992). The intense stromal reaction associated with pancreatic cancer (Mollenhauer et al., 1987) and its aggressive local invasive characteristics (Nagakawa et al., 1992), suggest that MMPs may play a role in the invasive phenotype of pancreatic cancer. This study reports the localisation of the protein products for MMP2, MMP3 and TIMP1 in these different types of cancer using immunohistochemistry.

### Materials and methods

### *Antibodies*

Four monoclonal murine antibodies were used that are specific to the 72 kDa type IV collagenase (MMP2, antibodies GL22 and GL8), stromelysin 1 (MMP3, antibody Mac78) and the tissue inhibitor of the metalloproteinases (TIMP1, antibody Mac15). These antibodies were raised against recombinant enzymes and subsequently characterised by Western blotting. They were compared with all members of the MMP/TIMP family to confirm specificity and absorption controls with recombinant enzymes performed

**Table I** Histopathology, stage and prognostically important factors of the sections used

| | *Adenocarcinoma pancreas* | *Ampullary adenocarcinoma* | *Low bile duct adenocarcinoma* | *Neuroendocrine tumours* |
|---|---|---|---|---|
| Number | 27 | 12 | 3 | 3 |
| TNM stage | | | | |
| T1 | 1 (4%) | 5 (42%) | 1 | 1 |
| T2 | 26 (96%) | 5 (42%) | 1 | 2 |
| T3 | 0 | 2 (17%) | 1 | 0 |
| N0 | 11 (41%) | 4 (33%) | 2 | 1 |
| N1 | 16 (59%) | 8 (67%) | 1 | 2 |
| Tumour differentiation | | | | |
| Well | 11[a] (41%) | 2[a] (17%) | 2 | 1 |
| Moderate | 10 (37%) | 3 (25%) | 0 | 1 |
| Poor | 6 (22%) | 7 (58%) | 1 | 1 |
| Tumour size (cm) | | | | |
| <2 | 3 (11%) | 5 (42%) | 1 | 1 |
| 2–5 | 22[b] (82%) | 4[b] (33%) | 1 | 2 |
| >5 | 2 (7%) | 1 (8%) | 1 | 0 |

[a]Pancreas cancers were better differentiated than ampullary cancers ($P<0.004$). [b]More pancreas cancers over 2 cm than ampullary cancers ($P<0.009$).

**Table II** Epithelial cell staining with the different antibodies

| *Antibody* | *Epithelial cell staining* | *Adenocarcinoma pancreas* | *Ampullary adenocarcinoma* | *Low bile duct adenocarcinoma* | *Neuroendocrine tumours* | *Chronic pancreatitis* |
|---|---|---|---|---|---|---|
| GL22 72 kDa Type IV collagenase (MMP2) | Negative | 0 | 0 | 0 | 2 | 0 |
| | Weak | 4 (15%) | 4 (33%) | 2 | 1 | 2 |
| | Moderate | 18 (67%) | 5 (42%) | 1 | 0 | 3 |
| | Strong | 5 (18%) | 3 (25%) | 0 | 0 | 0 |
| GL8 72 kDa Type IV collagenase (MMP2) | Negative | 0 | 0 | 0 | 2 | 0 |
| | Weak | 12 (44%) | 1 (8%) | 0 | 1 | 3 |
| | Moderate | 14 (52%) | 10 (83%) | 3 | 0 | 2 |
| | Strong | 1 (4%) | 1 (8%) | 0 | 0 | 0 |
| Mac 78 Stromelysin 1 (MMP3) | Negative | 2 (7%) | 1 (8%) | 1 | 1 | 0 |
| | Weak | 11 (41%) | 5 (42%) | 0 | 1 | 2 |
| | Moderate | 8 (30%) | 3 (25%) | 1 | 1 | 2 |
| | Strong | 5 (18%) | 3 (25%) | 1 | 0 | 1 |
| Mac 15 Tissue inhibitor of metalloproteinase (TIMP1) | Negative | 2 (7%) | 0 | 0 | 1 | 0 |
| | Weak | 21 (78%) | 8 (67%) | 2 | 2 | 4 |
| | Moderate | 4 (15%) | 4 (33%) | 1 | 0 | 1 |
| | Strong | 0 | 0 | 0 | 0 | 0 |

previously in our laboratories showed extinction of immunoreactivity (Afzal *et al.*, 1995). The antibodies were provided by the research and development section of Celltech UK and are not commercially available.

*Tissue sections*

All sections were part of the archival tissue collection of the Department of Histopathology, Royal Postgraduate Medical School, Hammersmith Hospital. All tissues had been fixed in 10% formalin and mounted in paraffin blocks from which 4 μm tissue sections were taken on to poly-L-lysine coated slides.

The murine avidin–biotin complex method was used for antigen detection with antibody dilutions of 1:100 for GL8 (stock solution 1.57 mg $ml^{-1}$ IgG), 1:40 for GL22 (stock solution 2.77 mg $ml^{-1}$ IgG), 1:40 for Mac78 (stock solution 2.88 mg $ml^{-1}$ IgG) and 1:25 for Mac15 (stock solution 1.75 mg $ml^{-1}$ IgG). Sections were dewaxed and rehydrated through ethanol, endogenous peroxidase was blocked with hydrogen peroxide and non-specific protein binding was reduced using goat serum. The primary antibody was applied at the correct dilution in a volume of 50–100 μl and left overnight at 4°C. The primary antibody was removed and the sections washed in phosphate-buffered saline (PBS), biotinylated anti-murine goat antibody at a dilution of 1:500 was applied to each section and left for 1 h (Dako, High Wycombe, UK). The sections were washed in PBS and streptavidin-labelled peroxidase at a dilution of 1:500 was applied to the sections and left for 1 h (Dako). The sections were then washed for a third time in PBS and developed in 500 ml of 0.01% PBS, 0.025%, 3.3′-diaminobenzidine tetrahydrochloride (Sigma, Poole, UK) and 500 μl of 30% hydrogen peroxide. The sections were washed again in PBS, counterstained using haematoxylin, dehydrated through alcohol and mounted in Pertex (Cellpath, UK).

Specificity of immunostaining was established using a negative control for each section, missing out the primary antibody and using 100 μl of the diluent alone. Also included in each batch of sections was a known positive control previously validated within the laboratory by parallel *in situ* hybridisation and/or enzymology.

Sections were taken from 50 individual patients: 27 with ductal adenocarcinoma of the pancreas, 12 with ampullary cancer, three with lower bile duct cancer, three with neuroendocrine pancreatic tumours and five with chronic pancreatitis (Table I). All sections were stained with each antibody and scored independently by two individuals (GWHS, SRB) for intensity of staining of epithelial, stromal and normal tissue. Data were collected using the following scoring system: negative, 0; weak, 1/2; moderate, 3/4; strong, 5/6. The results were compared with TNM staging, tumour grade and tumour size.

*Statistical analysis*

Statistical analysis was performed using the Pearson chi-squared test, the chi-squared test for trend and for other data using the Fisher's exact test where applicable (BMDP Statistical Software package and InStat by Graphpad).

### Results

The tumour and lymph node stage and tumour differentiation and size are shown in Table I. The intensity of staining in each tissue type with each antibody for epithelial, stromal and adjacent normal cells are shown in Tables II and III.

Immunoreactivities for MMP2, MMP3 and TIMP1 were greatest in adenocarcinomas of pancreas and ampulla compared with those in the other pathologies. None of the neuroendocrine tumours stained more than weakly with any

**Table III** Stromal cell staining with the different antibodies

| Antibody | Intensity of staining | Stromal cell staining: Adenocarcinoma pancreas | Stromal cell staining: Ampullary adenocarcinoma | Adjacent normal cell staining: Adenocarcinoma pancreas | Adjacent normal cell staining: Ampullary adenocarcinoma |
|---|---|---|---|---|---|
| GL22 72 kDa Type IV collagenase (MMP2) | Negative | 17 (63%) | 10 (83%) | 23 (85%) | 12 (100%) |
| | Weak | 7 (26%) | 2 (17%) | 3 (11%) | 0 |
| | Moderate | 3 (11%) | 0 | 1 (4%) | 0 |
| | Strong | 0 | 0 | 0 | 0 |
| GL8 72 kDa Type IV collagenase (MMP2) | Negative | 16 (59%) | 0 | 27 (100%) | 12 (100%) |
| | Weak | 9 (33%) | 7 (58%) | 0 | 0 |
| | Moderate | 2 (7%) | 5 (42%) | 0 | 0 |
| | Strong | 0 | 0 | 0 | 0 |
| Mac 78 Stromelysin1 (MMP3) | Negative | 22 (81%) | 8 (67%) | 25 (93%) | 10 (83%) |
| | Weak | 3 (11%) | 2 (17%) | 1 (4%) | 1 (8%) |
| | Moderate | 1 (4%) | 2 (17%) | 0 | 1 (8%) |
| | Strong | 0 | 0 | 0 | 0 |
| Mac 15 Tissue inhibitor or metalloproteinase (TIMP1) | Negative | 26 (96%) | 10 (83%) | 25 (93%) | 8 (67%) |
| | Weak | 1 (4%) | 2 (17%) | 2 (7%) | 3 (25%) |
| | Moderate | 0 | 0 | 0 | 1 (8%) |
| | Strong | 0 | 0 | 0 | 0 |

**Table IV** Epithelial cell staining with the antibody to TIMP1 in lymph node-positive and lymph node-negative patients

| | Lymph node status of patient | Intensity of epithelial cell staining with TIMP-1: Negative | Weak | Moderate | Strong |
|---|---|---|---|---|---|
| Pancreas cancer | Positive ($n$ = 16) | 1 (6%) | 14 (88%) | 1 (6%) | 0 |
| | Negative ($n$ = 11) | 1 (9%) | 7 (64%) | 3 (27%) | 0 |
| Ampullary cancer | Positive ($n$ = 8) | 0 | 7 (88%) | 1 (12%) | 0 |
| | Negative ($n$ = 4) | 0 | 1 (25%) | 3 (75%) | 0 |

**Table V** Epithelial cell staining with the antibody to MMP2 (GL8) according to tumour differentiation

| | *Tumour cell differentiation* | *Intensity of epithelial cell staining with MMP-2* Negative | Weak | Moderate | Strong |
|---|---|---|---|---|---|
| Pancreas cancer | Well ($n$ = 11) | 0 | 6* (55%) | 5* (45%) | 0 |
| | Moderate ($n$ = 10) | 0 | 5 (50%) | 4 (40%) | 1 (10%) |
| | Poor ($n$ = 6) | 0 | 0* | 6* (100%) | 0 |
| Ampullary cancer | Well ($n$ =2) | 0 | 0 | 2 (100%) | 0 |
| | Moderate ($n$ = 3) | 0 | 0 | 2 (67%) | 1 (33%) |
| | Poor ($n$ = 7) | 0 | 1 (14%) | 6 (86%) | 0 |

*$P<0.01$.

**Figure 1** A poorly differentiated ampullary adenocarcinoma invading around unstained benign tubular glands (with polarised nuclei). The tubules and single cells of the carcinoma show moderately intense staining by MMP3 (Mac 78) (ABC–immunoperoxidase, original magnification × 250).

**Figure 2** A poorly differentiated ductal adenocarcinoma of the pancreas showing moderate to strong heterogeneous cytoplasmic staining of the neoplastic cells for MMP3 (Mac 78). Weak staining of desmoplastic fibroblasts is also noted (ABC–immunoperoxidase, original magnification × 250).

of the antibodies, except MMP3, for which one tumour stained moderately. The pattern of staining in low bile duct tumours was similar to that in adenocarcinomas of the pancreas and ampulla.

All of the chronic pancreatitis sections showed immuno reactivity in acinar and ductal epithelial components with each antibody, which for MMP2 and MMP3 was at least moderately strong in three out of five, whereas for TIMP1 the epithelial staining was significant in only one out of five.

The immunoreactivity in the malignant epithelium was high with all of the antibodies in both adenocarcinomas of pancreas and ampulla (GL22 100% of pancreatic cases *vs* 100% of ampullary cases, GL8 100% *vs* 100%, Mac78 93% *vs* 92% and Mac15 93% *vs* 100%). In the malignant stromal tissue the immunoreactivity was much less (GL22 37% of pancreatic cases *vs* 17% of ampullary cases, GL8 40% *vs* 100%, Mac78 15% *vs* 34%, Mac15 4% *vs* 17%) and in normal tissue away from the malignant areas the immuno reactivity was even lower (GL22 15% of pancreatic cases *vs* 0% of ampullary cases, GL8 0% *vs* 0%, Mac78 4% *vs* 16%, Mac15 4% *vs* 33%) (Tables II and III).

With each antibody the epithelial cell staining was significantly stronger than either stromal or normal cell staining in both adenocarcinomas of the pancreas ($P<0.0001$) and ampulla ($P<0.003$) and with each antibody there was a significant trend for epithelial cells to have moderate to strong staining, stromal cells to have moderate to weak staining and normal tissue to be negative ($P<0.0001$).

There was strong correlation between the immunoreactivity of the two MMP2 antibodies ($P<0.0001$) and between MMP2 (GL22 or GL8) and MMP3 ($P<0.0001$). There was also correlation of expression between MMP2 (GL22 or GL8) and TIMP1 ($P<0.0001$) and between MMP3 and TIMP1 ($P<0.0001$), but this was only significant when the data were considered as two groups, negative compared with any level of immunoreactivity. The generally low levels of immunoreactivity of TIMP1 in the cancers and the small proportion of cases showing significant immunoreactivity justify this consideration.

Immunoreactivity for TIMP1 was absent or weak in 15 out of 16 cases of pancreatic cancer and four out of eight cases of ampullary cancer with lymph node metastases compared with eight out of 11 and one out of four cases respectively without lymph node metastases ($P<0.02$) (Table IV). There was increased immunoreactivity for MMP2 (GL8) with increasing tumour grade ($P<0.01$) (Table V).

There was no correlation or trend between lymph node stage or tumour differentiation and any of the other antibodies, nor between tumour stage and tumour size and any of the antibodies.

Figure 3 A well-differentiated ductal adenocarcinoma showing moderately intense cytoplasmic and apical membrane staining for MMP2 (GL22). Note the negative benign glands from the ampullary region (ABC–immunoperoxidase, original magnification × 250).

Figure 4 The same tumour as Figure 2, showing moderately strong cytoplasmic staining for TIMP1 (Mac 15) (ABC–immunoperoxidase, original magnification × 250).

Figure 5 A poorly differentiated ampullary adenocarcinoma showing strong staining for MMP2 (GL8), maximal in pleomorphic single invasive cells rather than neoplastic glands (ABC–immunoperoxidase, original magnification × 100).

### Discussion

This study has shown the reactivity of the two antibodies recognising MMP2 significantly correlated, reflecting their specificity. The immunoreactivity of MMP2 and MMP3 also correlated and this was perhaps surprising, as these two enzymes have different substrate specificities (Cottam and Rees, 1993) and appear to be important in different diseases. MMP3 plays a role in the activation of other members of the MMP family (Murphy *et al.*, 1987; Ito and Nagase, 1988) although not in the case of MMP2 where MT-MMP is a likely candidate (Sato *et al.*, 1994).

The antibodies to MMP2 (GL8 and GL22) both recognise pro-MMP2 and active MMP2 in solution phase, but in fixed tissues show a different reactivity pattern. GL22 demonstrates membrane and/or cytoplasmic staining which is polarised to the apex in differentiated cells, and rarely staining stromal cells while GL8 shows a cytoplasmic reactivity pattern in epithelial and stromal cells similar to other MMP2 antibodies that we have previously described (Poulsom *et al.*, 1992).

MMP2 has been shown to be produced by the RWP-1 human pancreatic cancer cell line (Zucker *et al.*, 1990). Plasma membrane fractions of the cell line were prepared by differential centrifugation and the membrane fraction extracted with *N*-butanol. Gelatin zymography showed proteinase bands of 92, 70 and 62 kDa and immunoblotting resulted in recognition of the 70 kDa protein but not the 92 kDa (Zucker *et al.*, 1990). It has been suggested that it is the membrane localisation of the MMP2 in the pancreatic cancer cells that is critical rather than the species of type IV collagenase (Zucker *et al.*, 1992). Certain human cancers have been shown to express MMP2, including adenocarcinoma of the colon, in which 10 out of 12 expressed the transcript for MMP2 but in desmoplastic fibroblasts rather than neoplastic cells. The same phenomenon was found in breast, skin and ovarian carcinomas (Poulsom *et al.*, 1992, 1993; Naylor *et al.*, 1994). The role of MMP3 in human adenocarcinoma is not well documented, although it appears to be important in the progression of squamous cell carcinomas (Ostrowski *et al.*, 1988).

While immunoreactivities of both MMP2 and MMP3

**Figure 6** Low-power view showing strong staining for MMP2 in poorly differentiated invasive ampullary adenocarcinoma cells, contrasting with the entrapped benign glands (ABC–immunoperoxidase, original magnification × 250).

(Figures 1 and 2) localised to the cytoplasm of the malignant epithelial cells in this series of pancreatic diseases, in many sections MMP2 (Figure 3) had large amounts of cellular membrane reactivity with the GL22 antibody especially at the invasion front. MMP2 appears to be produced in the stromal cells but activated and therefore localised by immunohistochemistry to the epithelial cells (Poulsom *et al*., 1992). The recent finding by Sato *et al*. (1994) of a new member of the matrix metalloproteinases which is membrane-bound (MT-MMP) and activates pro-MMP2 may help to explain these findings. There is no evidence that MMP3 is produced by stromal cells in the same way that MMP2 is, and this is confirmed by our findings of mainly cytoplasmic reactivity with this antibody. Although the specific inhibitor for MMP2 is TIMP2 (Stetler-Stevenson *et al*., 1990), TIMP1 will also bind MMP2 and elevated expression of TIMP1 transcripts was found in all five colorectal cancers examined in a recent series (Stetler-Stevenson *et al*., 1990). TIMP1 may be localised at the sites where MMP2 is active and this is supported by finding correlation of immunoreactivity for MMP2 and TIMP1 (Figures 3 and 4). The immunoreactivity of MMP3 and TIMP1 also correlated, in keeping with previous studies (Wilhelm *et al*., 1989).

It has been shown previously in human pancreatic cancer cells that expression of MMP1 correlated with spontaneous metastatic ability (Taniguchi *et al*., 1992). An increase in type I collagenolytic activity was noted in a subclone of the human pancreatic cancer cell line SUIT2 that demonstrated an increased metastasising ability in nude mice (Taniguchi *et al*., 1992). Reduced expression of TIMP is known to be associated with tumour cell line progression (Khokka *et al*., 1991) but evidence for this in human tumours has been lacking. A putative reduction in TIMP1 expression in patients with lymph node metastasis from other cancer types has not been previously described. During the progression of tumours the check on proteolytic activity afforded by the specific enzyme inhibitors may be reduced, which allows a relatively greater proteolytic activity of MMP2 or other metalloproteinases (Liotta and Stetler-Stevenson, 1991). The inverse relationship between tumour differentiation and expression of MMP2 provides further evidence of a central role for this enzyme in pancreatic and ampullary tumour progression.

These data strongly implicate MMP2, MMP3 and TIMP1 in pancreatic and ampullary carcinoma progression. MMP2 immunoreactivity was directly related to tumour dedifferentiation (Figures 5 and 6) and invasive potential and reduced expression of its specific inhibitor (TIMP1) may be involved with increased metastatic ability. Although adenocarcinomas of the pancreas and ampulla of Vater show distinct differences in clinical outcome the present study supports the notion that these differences may be related to the stage of clinical presentation or biological characteristics other than the expression of MMP2, MMP3 and TIMP1.

### References

AFZAL S, LALANI E-N, FOULKES W, CARDILLO M, BAKER T, PIGNATELLI M, DOCHERTY AJ AND STAMP GWH. (1995). Matrix metalloproteinase-2 expression in ovarian carcinomas and cell lines. *Lab. Invest*. (in press).

AGARWAL C, HEMBREE JR, RORKE EA AND ECKERT RL. (1994). Transforming growth factor beta 1 regulation of metalloproteinase production in cultured human cervical epithelial cells. *Cancer Res*., **54,** 943–949.

BASSET P, BELLOCQ JP, WOLF C, STOLL I, HUTIN P, LIMACHE JM, PODHAJCER OL, CHENARD MP, RIO MC AND CHAMBON P. (1990). A novel metalloproteinase gene specifically expressed in stromal cells of breast carcinomas. *Nature*, **348,** 699–704.

BISWAS C. (1982). Tumor cell stimulation of collagenase production by fibroblasts. *Biochem. Biophys. Res. Comm*., **109,** 1026–1034.

BROWN PD, LEVY AT, MARGULIES IM, LIOTTA LA AND STETLER-STEVENSON WG. (1990). Independent expression and cellular processing of Mr 72,000 type IV collagenase and interstitial collagenase in human tumorigenic cell lines. *Cancer Res*., **50,** 6184–6191.

CHARPIN C, LISSITZKY JC, JACQUEMIER J, LAVAUT MN, KOPP F, POURREAU-SCHNEIDER N, MARTIN PM AND TOGA M. (1986). Immunohistochemical detection of laminin in 98 human breast carcinomas: A light and electron microscopic study. *Hum. Pathol*., **17,** 355–365.

CHIN JR, MURPHY G AND WERB Z. (1985). Stromelysin, a connective tissue-degrading metalloendopeptidase secreted by stimulated rabbit synovial fibroblasts in parallel with collagenase. *J. Biol. Chem*., **260,** 12367–12376.

COTTAM DW AND REES RC. (1993). Regulation of matrix metalloproteinases: their role in tumor invasion and metastasis (review). *Int. J. Oncol*., **2,** 861–872.

DECLERCK YA, YEAN T-D, RATZKIN BJ, LU HS AND LANGLEY KE. (1989). Purification and characterisation of two related but distinct metalloproteinase inhibitors secreted by bovine aortic endothelial cells. *J. Biol. Chem*., **264,** 17445–17453.

FORSTER SJ, TALBOT IC, CLAYTON DG AND CRITCHLEY DR. (1986). Tumour basement membrane laminin in adenocarcinoma of rectum: an immunohistochemical study of biological and clinical significance. *Int. J. Cancer*, **37,** 813–817.

GRAY ST, YUN K, MOTOORI T AND KUYS YM. (1993). Interstitial collagenase gene expression in colonic neoplasia. *Am. J. Pathol*., **143,** 663–671.

HADDOCK G AND CARTER DC. (1990). Aetiology of pancreatic cancer. *Br. J. Surg*., **77,** 1159–1166.

HAMDY FC, FADLON EJ, COTTAM D, LAWRY J, THURRELL W, SILCOCKS PB, ANDERSON JB, WILLIAMS JL AND REES RC. (1994). Matrix metalloproteinase 9 expression in primary human prostatic adenocarcinoma and benign prostatic hyperplasia. *Br. J. Cancer*, **69,** 177–182.

HART IR, GOODE NT AND WILSON RE. (1989). Molecular aspects of the metastatic cascade. *Biochim. Biophys. Acta*, **989,** 65–84.

HEWITT RE, LEACH IH, POWE DG, CLARK IM, CAWSTON TE AND TURNER DR. (1991). Distribution of collagenase and tissue inhibitor of metalloproteinases (TIMP) in colorectal tumours. *Int. J. Cancer*, **49,** 666–672.

ITO N AND NAGASE H. (1988). Evidence that human rheumatoid synovial matrix metalloproteinase 3 is an endogenous activator of procollagenase. *Arch. Biochem. Biophys*., **267,** 211–216.

KHOKKA R, WATERHOUSE P, LALA P, ZIMMER M AND DENHARDT DT. (1991). Increased proteinase expression during tumour progression of tissue inhibitor of metalloproteinases cell lines down-modulated for levels: a new transformation paradigm? *J. Cancer Res. Clin. Oncol.*, **117**, 333–338.

LEE CS, MONTEBELLO J, GEORGIOU T AND RODE J. (1994). Distribution of type IV collagen in pancreatic adenocarcinoma and chronic pancreatitis. *Int. J. Exp. Pathol.*, **75**, 79–83.

LIOTTA LA AND STETLER-STEVENSON WG. (1991). Tumor invasion and metastasis: an imbalance of positive and negative regulation. *Cancer Res.*, **51** (suppl.), 5054s–5059s.

LIOTTA LA, TRYGGVASON K, GARBISA S, HART I, FOLTZ CM AND SHAFIE S. (1980). Metastatic potential correlates with enzymatic degradation of basement membrane collagen. *Nature*, **284**, 67–68.

MATRISIAN LM, McDONNELL S, MILLER DB, NAVRE M, SEFTOR EA AND HENDRIX MJC. (1991). The role of the matrix metalloproteinase stromelysin in the progression of squamous cell carcinomas. *Am. J. Med. Sci.*, **302**, 157–162.

MIYAZAKI K, HATTORI Y, UMENISHI F, YASUMITSU H AND UMEDA M. (1990). Purification and characterization of extracellular matrix-degrading metalloproteinase, matrin (pump-1), secreted from human rectal carcinoma cell line. *Cancer Res.*, **50**, 7758–7764.

MOLL UM, LANE B, ZUCKER S, SUZUKI K AND NAGASE H. (1990). Localisation of collagenase at the basal plasma membrane of a human pancreatic carcinoma cell line. *Cancer Res.*, **50**, 6995–7002.

MOLLENHAUER J, ROETHER I AND KERN HF. (1987). Distribution of extracellular matrix proteins in pancreatic ductal adenocarcinoma and its influence on tumour cell proliferation *in vitro*. *Pancreas*, **2**, 14–24.

MULLER D, QUANTIN B, GESNEL M-C, MILLON-COLLARD R, ABECASSIS J AND BREATHNACH R. (1988). The collagenase gene family in humans consists of at least four members. *Biochem. J.*, **253**, 187–192.

MURPHY G, COCKETT MI, STEPHENS PE, SMITH BJ AND DOCHERTY AJP. (1987). Stromelysin is an activator of procollagenase. *Biochem. J.*, **248**, 265–268.

MURPHY G, COCKETT MI, WARD RV AND DOCHERTY AJ. (1991*a*). Matrix metalloproteinase degradation of elastin, type IV collagen and proteoglycan. A quantitative comparison of the activities of 95 kDa and 72 kDa gelatinases, stromelysins-1 and -2 and punctuated metalloproteinase (PUMP). *Biochem. J.*, **277**, 277–279.

MURPHY GJP, MURPHY G AND REYNOLDS JJ. (1991*b*). The origin of matrix metalloproteinases and their familial relationships. *Febbs Lett.*, **289**, 4–7.

NAGAKAWA T, KONISHI I, HIGASHINO Y, UENO K, OHTA T, KAYAHARA M, UEDA N, MAEDA K AND MIYAZAKI I. (1989). The spread and prognosis of carcinoma in the region of the pancreatic head. *Jpn. J. Surg.*, **19**, 510–518.

NAGAKAWA T, KAYAHARA M, UENO K, OHTA T, KONISHI I, UEDA N AND MIYAZAKI I. (1992). A clinicopathologic study on neural invasion in cancer of the pancreatic head. *Cancer*, **69**, 930–935.

NAYLOR MS, STAMP GW, DAVIES BD AND BALKWILL FR. (1994). Expression and activity of MMPs and their regulators in ovarian cancer. *Int. J. Cancer*, **58**, 50–56.

NEOPTOLEMOS JP, TALBOT IC, SHAW DC AND CARR-LOCKE DL. (1988). Long-term survival after resection of ampullary carcinoma is associated independently with tumour grade and a new staging classification that assesses local invasiveness. *Cancer*, **61**, 1403–1407.

OKADA Y, TSUCHIYA H, SHIMIZU H, TOMITA K, NAKANISHI I, SATO H, SEIKI M, YAMASHITA K AND HAYAKAWA T. (1990). Induction and stimulation of 92-KDa gelatinase/type IV collagenase production in osteosarcoma and fibrosarcoma cell lines by tumor necrosis factor α. *Biochem. Biophys. Res. Comm.*, **171**, 610–617.

OKADA Y, GONOJI Y, NAKA K, TOMITA K, NAKANISHI I, IWATA K, YAMASHITA K AND HAYAKAWA T. (1992). Matrix metalloproteinase 9 (92-kDa gelatinase/type IV collagenase) from HT 1080 human fibrosarcoma cells. Purification and activation of the precursor and enzymic properties. *J. Biol. Chem.*, **267**, 21712–21719.

OSTROWSKI LE, FINCH J, KRIEG P, MATRISIAN L, PATSKAN G, O'CONNELL JF, PHILLIPS J, SLAGA TJ, BREATHNACH R AND BOWDEN GT. (1988). Expression pattern of a gene for a secreted metalloproteinase during late stages of tumor progression. *Mol. Carcinogenesis*, **1**, 13–19.

OVERALL CM, WRANA JL AND SODEK J. (1991). Transcriptional and post-transcriptional regulation of 72-kDa gelatinase/type IV collagenase by tranforming growth factor-beta 1 in human fibroblásts. Comparisons with collagenase and tissue inhibitor of matrix metalloproteinase gene expression. *J. Biol. Chem.*, **266**, 14064–14071.

PONTON A, COULOMBE B AND SKUP D. (1991). Decreased expression of tissue inhibitor of metalloproteinases in metastatic tumor cells leading to increased levels of collagenase activity. *Cancer Res.*, **51**, 2138–2143.

POULSOM R, PIGNATELLI M, STETLER-STEVENSON WG, LIOTTA LA, WRIGHT PA, JEFFREY RE, LONGCROFT JM, ROGERS L AND STAMP GWH. (1992). Stromal expression of 72KDa type IV collagenase (MMP-2) and TIMP-2 mRNAs in colorectal neoplasia. *Am. J. Pathol.*, **141**, 389–396.

POULSOM R, HANBY AM, PIGNATELLI M, JEFFERY RE, LONGCROFT JM, ROGERS L AND STAMP GWH. (1993). Expression of gelatinase A and TIMP-2 mRNAs in desmoplastic fibroblasts in both mammary carcinomas and basal cell carcinomas of the skin. *J. Clin. Pathol.*, **46**, 429–436.

PYKE C, RALFKIAER E, HUHTALA P, HURSKAINEN T, DANO K AND TRYGGVASON K. (1992). Localisation of messenger RNA for $M_r$ 72,000 and 92,000 type IV collagenases in human skin cancers by *in situ* hybridisation. *Cancer Res.*, **52**, 1336–1341.

RUSSELL RC. (1990). Surgical resection for cancer of the pancreas. *Baillière's Clin. Gastroenterol.*, **4**, 889–916.

SATO H, TAKINO T, OKADA Y, CAO J, SHINAGAWA A, YAMAMOTO E AND SEIKI M. (1994). A matrix metalloproteinase expressed on the surface of invasive tumour cells. *Nature*, **370**, 61–65.

SHIMA I, SASAGURI Y, KUSUKAWA J, NAKANO R, YAMANA H, FUJITA H, KAKEGAWA T AND MORIMATSU M. (1993). Production of matrix metalloproteinase 9 (92-kDa gelatinase) by human oesophageal squamous cell carcinoma in response to epidermal growth factor. *Br. J. Cancer*, **67**, 721–727.

STETLER-STEVENSON WG, BROWN PD, ONISTO M, LEVY AT AND LIOTTA LA. (1990). Tissue inhibitor of metalloproteinases-2 (TIMP-2) mRNA expression in tumor cell lines and human tumor tissues. *J. Biol. Chem.*, **265**, 13933–13938.

TANIGUCHI S, IWAMURA T AND KATSUKI T. (1992). Correlation between spontaneous metastatic potential and type 1 collagenolytic activity in a human pancreatic cell line (SUIT-2) and sublines. *Clin. Exp. Metastasis*, **10**, 259–266.

UNEMORI EN, FERRARA N, BAUER EA AND AMENTO EP. (1992). Vascular endothelial growth factor induces interstitial collagenase expression in human endothelial cells. *J. Cell Physiol.*, **153**, 557–562.

URBANSKI SJ, EDWARDS DR, HERSHFIELD N, HUCHCROFT SA, SHAFFER E, SUTHERLAND L AND KOSSAKOWSKA AE. (1993). Expression pattern of metalloproteinases and their inhibitors changes with the progression of human sporadic colorectal neoplasia. *Diagn. Mol. Pathol.*, **2**, 81–89.

URIA JA, FERRANDO AA, VELASCO G, FREIJE JMP AND LOPEZ-OTIN C. (1994). Structure and expression in breast tumours of human TIMP-3, a new member of the metalloproteinase inhibitor family. *Cancer Res.*, **54**, 2091–2094.

WELGUS HG AND STRICKLIN GP. (1983). Human skin fibroblast collagenase inhibitor. *J. Biol. Chem*, **258**, 12259–12264.

WELGUS HG, JEFFREY JJ, EISEN AZ, ROSWIT WT AND STRICKLIN GP. (1985). Human skin fibroblast collagenase: interaction with substrate and inhibitor. *Collagen Rel. Res.*, **5**, 167–179.

WILHELM SM, COLLIER IE, MARMER BL, EISEN AZ, GRANT GA AND GOLDBERG GI. (1989). SV40-transformed human lung fibroblasts secrete a 92-kDa type IV collagenase which is identical to that secreted by normal human macrophages. *J. Biol. Chem.*, **264**, 17213–17221.

YAMAGUCHI K AND ENJOJI M. (1989). Carcinoma of the pancreas: a clinicopathologic study of 96 cases with immunohistochemical observations. *Jpn. J. Clin. Oncol.*, **19**, 14–22.

YOSHIMOTO M, ITOH F, YAMAMOTO H, HINODA Y, IMAI K AND YACHI A. (1993). Expression of MMP-7(PUMP-1) mRNA in human colorectal cancers. *Int. J. Cancer*, **54**, 614–618.

ZUCKER S, MOLL UM, LYSIK RM, DIMASSIMO EI, STETLER-STEVENSON WG, LIOTTA LA AND SCHWEDES JW. (1990). Extraction of type-IV collagenase/gelatinase from plasma membranes of human cancer cells. *Int. J. Cancer*, **45**, 1137–1142.

ZUCKER S, MOLL UM, LYSIK RM, DIMASSIMO EI, SCHWEDES JW AND LIOTTA LA. (1992). Extraction of type IV collagenase/gelatinase from plasma membranes of human pancreatic cancer cells. *Matrix Suppl.*, **1**, 411.